# Strain-Induced 2H to 1T′ Phase Transition in Suspended MoTe_2_ Using Electric Double Layer Gating

**DOI:** 10.1021/acsnano.3c04701

**Published:** 2023-11-10

**Authors:** Shubham Sukumar Awate, Ke Xu, Jierui Liang, Benjamin Katz, Ryan Muzzio, Vincent H. Crespi, Jyoti Katoch, Susan K. Fullerton-Shirey

**Affiliations:** † Department of Chemical and Petroleum Engineering, University of Pittsburgh, Pittsburgh, Pennsylvania 15260, United States; ‡ School of Physics and Astronomy, Rochester Institute of Technology, Rochester, New York 14623, United States; ¶ Microsystems Engineering, Rochester Institute of Technology, Rochester, New York 14623, United States; § Department of Physics, The Pennsylvania State University, University Park, Pennsylvania 16802, United States; ∥ Department of Physics, Carnegie Mellon University, Pittsburgh, Pennsylvania 15213, United States; ⊥ Department of Materials Science and Engineering, The Pennsylvania State University, University Park, Pennsylvania 16802, United States; # Department of Chemistry, The Pennsylvania State University, University Park, Pennsylvania 16802, United States; @ Department of Electrical and Computer Engineering, University of Pittsburgh, Pittsburgh, Pennsylvania 15260, United States

**Keywords:** 2D material, semiconductor−metal transition, strain, electric double layer, single-ion conductor

## Abstract

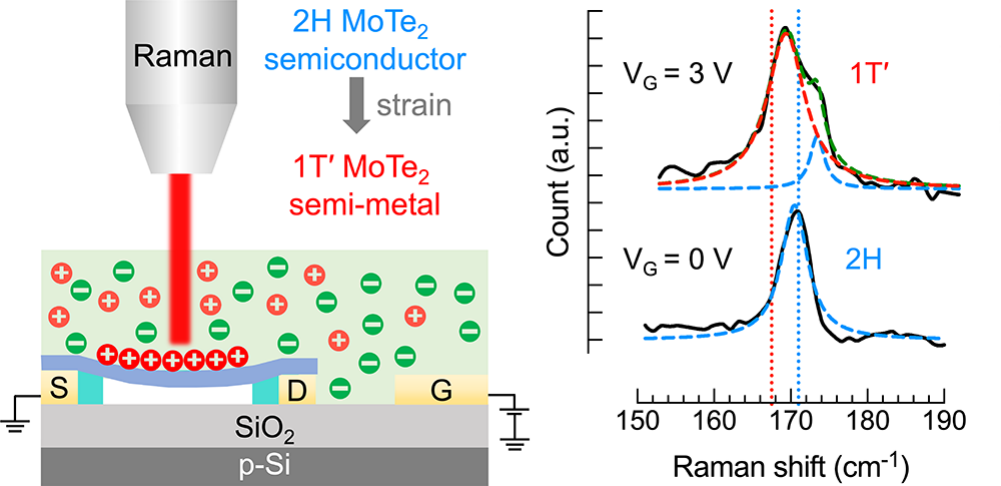

MoTe_2_ can be converted from the semiconducting (2H) phase to the semimetallic (1T′) phase by several stimuli including heat, electrochemical doping, and strain. This type of phase transition, if reversible and gate-controlled, could be useful for low-power memory and logic. In this work, a gate-controlled and fully reversible 2H to 1T′ phase transition is demonstrated via strain in few-layer suspended MoTe_2_ field effect transistors. Strain is applied by the electric double layer gating of a suspended channel using a single ion conducting solid polymer electrolyte. The phase transition is confirmed by simultaneous electrical transport and Raman spectroscopy. The out-of-plane vibration peak (A_1g_)—a signature of the 1T′ phase—is observed when *V*_SG_ ≥ 2.5 V. Further, a redshift in the in-plane vibration mode (E_2g_) is detected, which is a characteristic of a strain-induced phonon shift. Based on the magnitude of the shift, strain is estimated to be 0.2–0.3% by density functional theory. Electrically, the temperature coefficient of resistance transitions from negative to positive at *V*_SG_ ≥ 2 V, confirming the transition from semiconducting to metallic. The approach to gate-controlled, reversible straining presented here can be extended to strain other two-dimensional materials, explore fundamental material properties, and introduce electronic device functionalities.

## Introduction

The polymorphs of transition metal dichalcogenides (TMDs) exhibit distinct electronic transport properties. For example, the polymorphs of MoX_2_ and WX_2_ (X = S, Se, Te) include the semiconducting 2H phase, metallic 1T phase, and semimetallic 1T′ phase.^1^ Phase engineering of these materials has potential use in electrocatalysis,^2^ nonlinear optics,^3^ electronics and optoelectronics,^4−7^ ferroelectricity,^8^ and superconductivity.^9^ In catalysis, for example, 1T′ MoS_2_ shows enhanced hydrogen evolution compared to the 2H phase.^10^ In electronics, the carrier mobility of a MoTe_2_ transistor can be increased by a factor of about 50 by creating an in-plane ohmic junction between 2H and 1T′ phases.^7^ Among the TMDs, MoTe_2_ has the lowest reported potential energy difference between the semiconducting 2H and semimetallic 1T′ phases (40 meV).^11,12^ This positions MoTe_2_ as the leading 2D candidate for devices such as low-power phase-change memory and transistors.^6,7^

**Figure 1:**
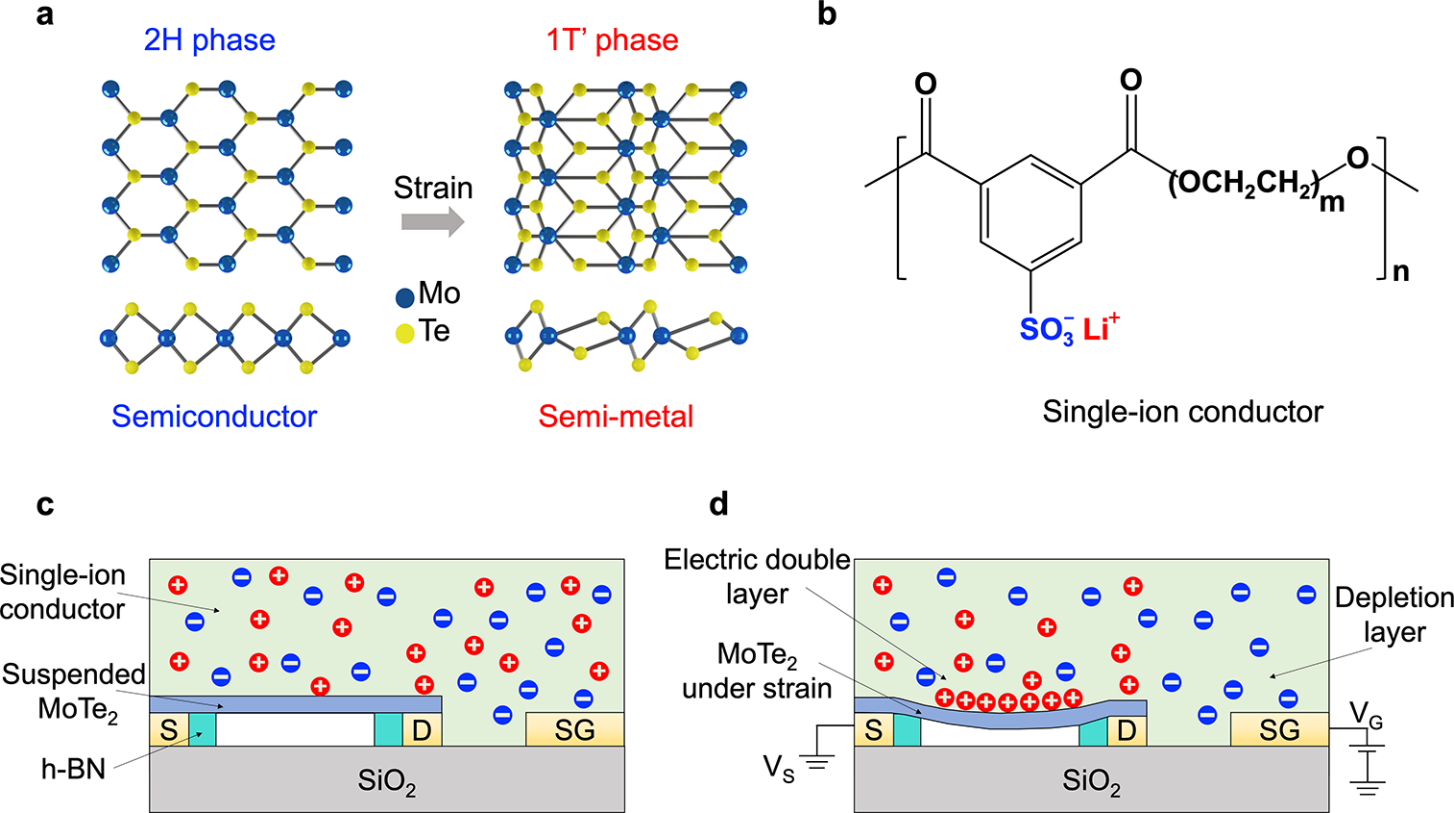
Molecular structures, strain mechanism, and device structure of a suspended MoTe_2_ FET gated by a single-ion conductor. (a) Top and side views of 2H and 1T′ crystal phases of MoTe_2_; 2H coordination is trigonal prismatic and 1T′ is distorted octahedral. (b) Molecular structure of the single-ion conductor (PE900) containing an anion (SO_3_^–^) covalently bonded to the polymer backbone and a cation (Li^+^) that can freely move in response to an electric field. Schematic of a single-ion conductor-gated, suspended MoTe_2_ FET (c) without and (d) with a positive gate voltage applied. The charge imbalance creates stress at the electrolyte/MoTe_2_ interface that results in longitudinal strain of the flake. The flake bends inside the cavity, and the crystal transitions from the 2H to the 1T′ phase.

The crystal structures of the 2H and 1T′ phases of MoTe_2_ are illustrated in Figure 1(a). The 2H phase has a trigonal prismatic structure, whereas the metallic 1T and semimetallic 1T′ phases have octahedral and distorted octahedral structures, respectively. In the 1T′ structure, the decreased distance between Mo atoms increases their d-orbital overlap, resulting in semimetallic conduction.^13,14^ Previous reports showed that the semiconducting 2H phase can be transformed to 1T′ by heat treatment,^15^ phase patterning via lasing,^7^ alloying,^16^ electrochemical doping,^12^ electron diffusion from a 2D electride,^17^ and strain.^18−20^ Thermal approaches use heat to evaporate Te atoms at high temperatures (e.g., *T* > 800 °C), creating Te vacancies. Density functional theory (DFT) predicts that creating >2% Te vacancies in 2H-MoTe_2_ will lead to a stable 1T′ phase.^21^ Zakhidov et al. reported a room-temperature, partially reversible electrochemical transition, where the phase change was mediated by the generation of Te vacancies.^12^ Among the reported techniques, most are irreversible or partially reversible, making it challenging to implement the mechanism for memory applications.^7,12,15,17^

In contrast to approaches that require altering the TMD composition, strain-based mechanisms are promising to achieve a fully reversible 2H to 1T′ phase transition, because they do not require the insertion or removal of atoms. Duerloo et al.^11^ predicted 0.3–3% as the range of a tensile strain required to transform MoTe_2_ from 2H to 1T′ under uniaxial conditions at room temperature. Experimentally, Song et al.^18^ used an atomic force microscopy (AFM) tip to apply 0.2% tensile strain on a suspended MoTe_2_ flake and confirmed a reversible phase transition. Other reports demonstrated the reversible phase transition by applying mechanical strain (∼0.2%) using a ferroelectric or piezoelectric substrate with an applied electric field.^6,22^ However, straining single-crystal ferroelectrics over 0.25–0.3 mm requires voltage pulses greater than 100 V.^6^ These efforts have significantly advanced our understanding of 2D materials and devices via strain engineering; however, approaches that require locally straining a device with an AFM tip or globally straining all devices using ferroelectric or piezoelectric substrates with large gate voltages (>10 V)^6,22,23^ are not practical for large-scale device integration. What is needed is an approach to reversibly strain individual devices at lower voltage via the field effect without straining neighboring devices.

Electric double layer (EDL) gating is a field effect technique in which ions in an electrolyte are used to control carrier transport in a semiconducting material.^24^ The main advantage of EDL gating is that it generates large capacitance densities (∼10–100 μF cm^–2^) that are an order of magnitude higher than conventional gate dielectrics.^25^ However, “dual-ion conductors” (i.e., those with both mobile cations and anions) are typically used for gating semiconductors, and the identical charge distribution at both double layers (one at the electrolyte/semiconductor interface and the other at the electrolyte/gate interface) does not produce significant strain. In contrast, ion-based electroactive polymers change shape in response to an applied electric field due to a charge imbalance created by ions. These materials have been explored for their use in artificial muscles, actuators, and sensors.^26−28^ Charge imbalance is achieved by allowing one type of ion to remain mobile while immobilizing the other, giving rise to the name “single-ion conductor”. At the microscale, stress is induced in the polymer backbone by the electrostatic repulsion of the local, unbalanced net charge.^29,30^ Specifically, under an applied electric field, mobile ions accumulate to form an EDL on one side, which expands the polymer via repulsion, while the depletion layer on the other side contracts. The physics describing the electromechanical transduction of single-ion conductors has been reported by Lee et al., where the voltage-dependent induced charge is coupled to the resulting curvature.^31^

Here, we employ a single-ion conductor to achieve field-induced, localized strain that reversibly transforms the semiconducting 2H phase of MoTe_2_ to the semimetallic 1T′ phase. The device is a few-layer suspended MoTe_2_ field effect transistor (FET) gated by a custom-synthesized, polyester-based single-ion conductor. The electrical and structural properties of the suspended FETs are measured simultaneously by applying voltage and measuring current while collecting Raman spectra; the transition from 2H to 1T′ is confirmed at *V*_SG_ > 2.5 V. Specifically, the shift of the in-plane vibration mode to lower wavenumbers confirms lateral strain, and DFT calculations estimate the strain to be 0.2–0.3%, consistent with previously reported values. Raman mapping indicates that the phase change mainly occurs in the suspended region. The transition is also confirmed by electrical measurements. Output characteristics show a ∼2000× decrease in resistance for *V*_SG_ > 1.5 V. Temperature-dependent sheet resistance measurements reveal a change in the temperature coefficient of resistance from negative to positive at *V*_SG_ ≥ 2 V, confirming the transition from semiconducting to metallic conduction. This work demonstrates that a single device can be strained using field effects and without inducing electrochemistry by relying on the electrostatic imbalance induced by a single-ion-conducting, solid polymer electrolyte.

## Results and Discussion

The single-ion conductor is a polyester of dicarboxylic 5-sulfoisophthalate lithium with a poly(ethylene glycol) spacer of 900 repeat units. The structure of the polymer, abbreviated PE900-Li, is illustrated in Figure 1(b) and was synthesized as described by Dou et al.^32^ The anion (SO_3_^–^) is covalently bonded to the polymer backbone, while the cation (Li^+^) is free to move throughout the polymer under an applied electric field. When no gate bias is applied, cations and anions are uniformly distributed across the electrolyte (Figure 1(c)). When positive gate bias is applied, Li^+^ ions migrate to form an EDL at the single-ion conductor/MoTe_2_ interface. The anions are immobile, and therefore a cationic depletion layer will be created near the gate/single-ion conductor interface^33^ (Figure 1(d)). We previously used this single-ion conductor to EDL-gate *supported* graphene and MoTe_2_ FETs (meaning that the channel is entirely supported by the substrate) and observed a suppression of the *p*-branch as would be expected for stationary anions.^34^ Based on the mechanism by which strain is induced in ion-based electroactive polymers (EAPs),^31^ we hypothesize that the *suspended* 2H phase MoTe_2_ can be transformed to the 1T′ phase by EDL gating using the single-ion conductor.

**Figure 2:**
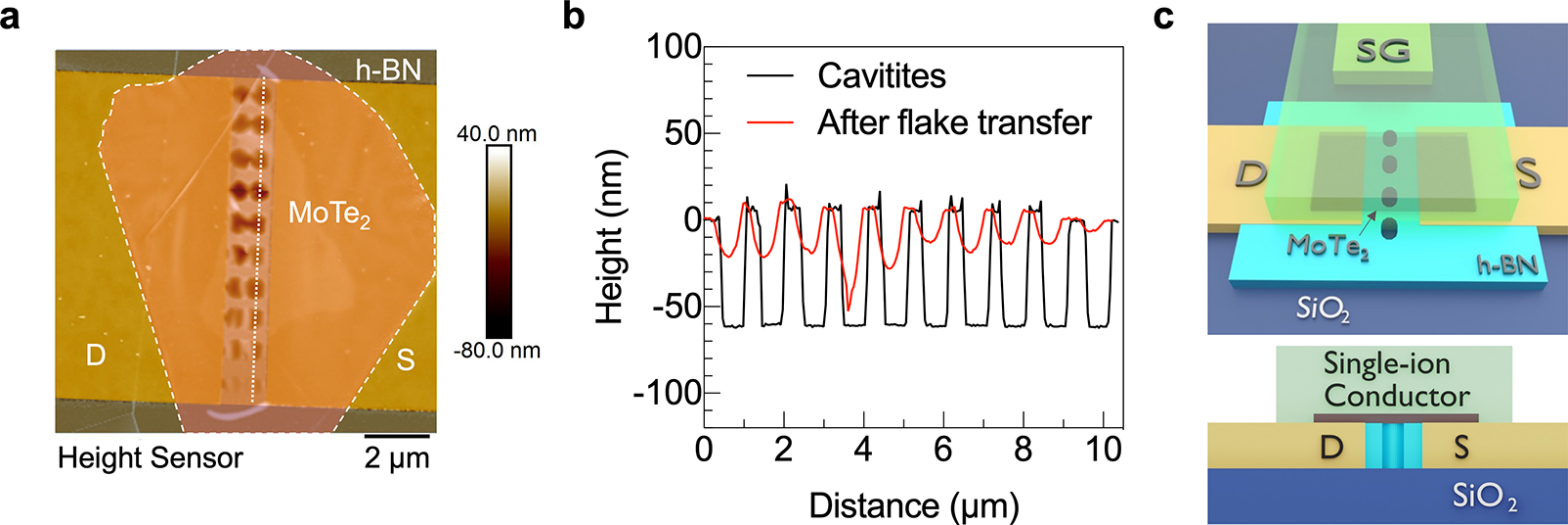
Characterization of the suspended MoTe_2_ FET gated by a single-ion conductor. (a) AFM topography scan of the suspended MoTe_2_ FET. The MoTe_2_ flake is ∼7.5 nm thick (Supporting Information Part 2) and outlined by the solid dashed line. The S/D contacts are buried in h-BN by etching the h-BN and evaporating 5 nm Ti and ∼55 nm Au. (b) Cavity depth before and after the MoTe_2_ transfer, confirming suspension (c) Schematics of the suspended MoTe_2_ FET side-gated using a single-ion conductor; the bottom cross-sectional view bisects one of the cavities.

The EDL-gated suspended FET uses a lateral side gate geometry. To ensure the MoTe_2_ is lying flat across its entire area and there is no unregulated strain, source/drain contacts are buried in the hexagonal boron nitride (h-BN) substrate. Specifically, 50–60 nm thick h-BN was mechanically exfoliated on 90 nm SiO_2_. E-beam lithography was used to define the shape to be removed from h-BN for the buried source/drain contacts followed by reactive ion etching (O_2_ plasma). The height of the etched region was characterized using AFM, and the contact metal (Ti/Au) was deposited in the etched region to match the height of the h-BN substrate. The flakes used to fabricate suspended FETs are typically a few nanometers thick, which is ∼3 orders of magnitude smaller than the lateral size of the flakes (few μm). We observe that flakes tend to sag into cavities larger than 1 μm; therefore, to achieve a complete channel suspension, the maximum lateral size of the cavity is optimized to be 500 nm. Multiple cavities were fabricated between source/drain contacts to maximize the flake suspension while supporting the channel at regular intervals. Cavities were patterned using e-beam lithography and etched using reactive-ion etching (O_2_ plasma). Few-layer (5–7 nm) 2H-MoTe_2_ flakes were dry transferred using a polycarbonate/polydimethylsiloxane (PC/PDMS) stamp. Figure 2(a) is the topological AFM scan of the device after transfer, showing the flake situated on the source/drain contacts and suspended over the cavities. The suspension of the flake is confirmed by a line scan along the cavities before and after the flake transfer (Figure 2(b)). The height of the cavities decreases from 60 nm to 20 nm, averaged over 20 cavities, confirming a 40 nm suspension distance. (Note that the appearance of sagging in the AFM image is exaggerated because the length scales of the *x* and *y* axes differ by 3 orders of magnitude.) The last step is the deposition of PE900-Li (3 wt % in dimethylformamide (DMF)) in an Ar-filled glovebox followed by natural evaporation to achieve a solid film. A 3D schematic of the final device is depicted in Figure 2(c), with details of the fabrication process and flake characterization reported in Supporting Information Parts 1 and 2.

**Figure 3:**
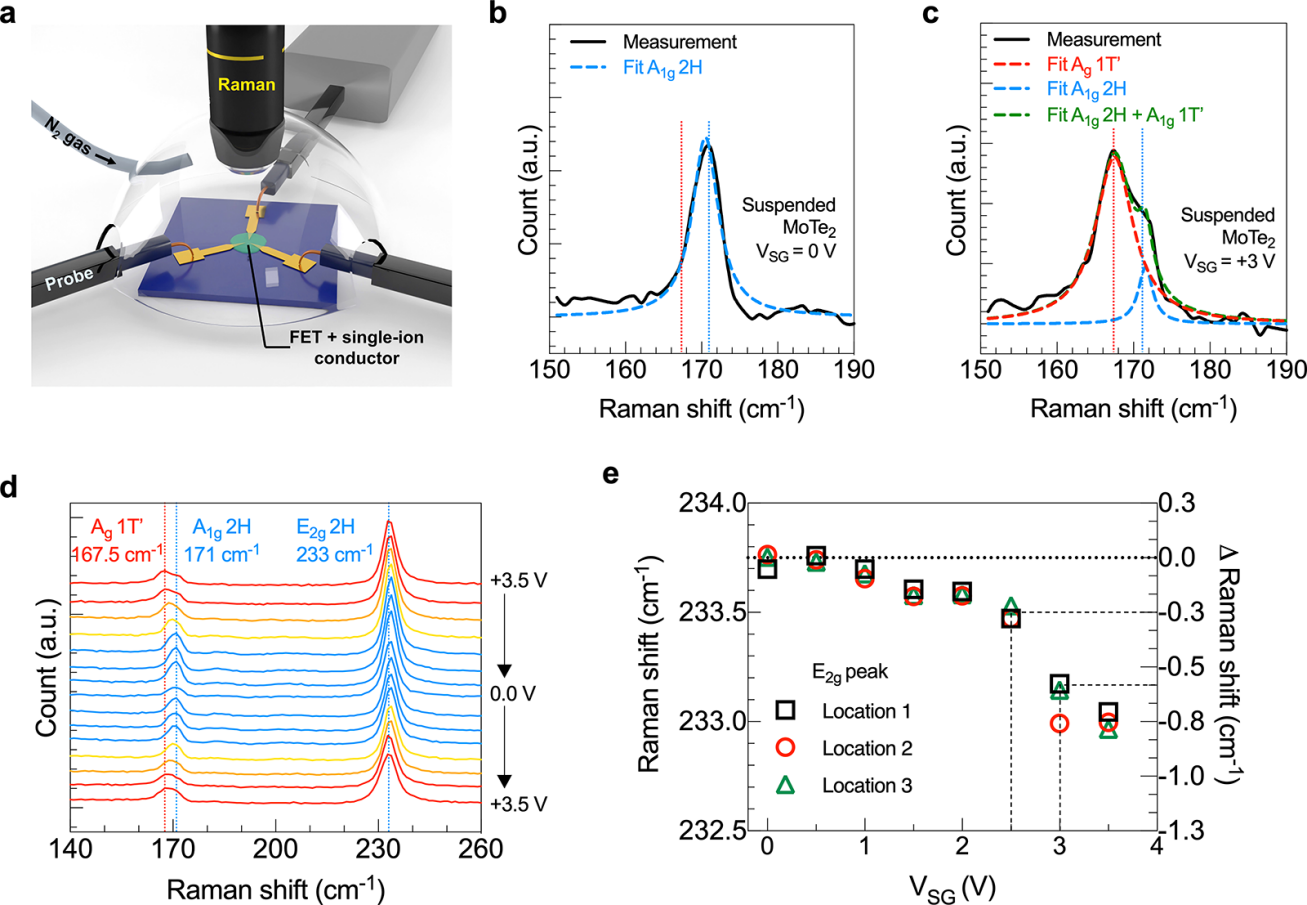
Raman spectroscopy. (a) Schematic of the combined electrical and Raman spectroscopy setup. Measured Raman spectrum (solid black line) of the out-of-plane vibration mode of a suspended MoTe_2_ FET gated using a single-ion conductor at (b) *V*_SG_ = 0 V and (c) *V*_SG_ = 3 V. Dashed lines represents Lorentzian fitting of the peak. Vertical dotted lines on *x* axes represent the expected positions for 2H (blue) and 1T′ (red) phases. (d) Raman spectra when *V*_SG_ is varied from +3.5 to 0 to +3.5 V in the interval of 0.5 V. (e) Position of the in-plane vibration mode (E_2g_) peak as a function of *V*_SG_. Right *y* axis represents the redshift in the peak position relative to *V*_SG_ = 0 V, i.e., Δ Raman shift.

Next, Raman spectroscopy is used to detect the phase transition in the EDL-gated, suspended MoTe_2_ device. The 2H phase has distinct in- and out-of-plane vibration modes in Raman spectra at wavenumbers of 233 (E_2g_) and 171 (A_1g_) cm^–1^, respectively.^35,36^ For the 1T′ phase, the in-plane vibration mode is absent because the crystal structure is distorted; therefore, only an out-of-plane vibration peak can be detected at 167.5 (A_g_) cm^–1^. Neither SiO_2_ or the single-ion conductor contributes to the MoTe_2_ spectrum, as shown in the Supporting Information Parts 3 and 4. A home-built measurement system, depicted in Figure 3(a), was used to collect the Raman spectra, while the *V*_SG_ was modulated between −3 and +3 V and a constant *V*_DS_ of 0.5 V was applied. To protect the single-ion conductor from moisture, N_2_ gas was flowed around the device during the measurements.

When no gate voltage is applied, the 2H phase is confirmed by the characteristic 171 and 233 cm^–1^ (2H) peaks associated with out-of-plane and in-plane vibrations, respectively. Figure 3(b) shows the measured out-of-plane 171 cm^–1^ peak and the Lorentzian fit, confirming the peak position. This result also indicates that the slight sagging of the suspended flake shown in Figure 2(b) does not induce significant strain because the 2H phase is maintained. Further confirmation of the absence of built-in strain is the equivalent position of the strain-sensitive E_2g_ mode in both suspended MoTe_2_ and supported MoTe_2_ at a *V*_SG_ of 0 V (Supporting Information part 5). When *V*_SG_ is increased to 3 V, the measured spectrum consists of a mixture of two peaks: one of higher intensity at 167.5 cm^–1^ and one of lower intensity at 171 cm^–1^ (Figure 3(c)). The new peak at 167.5 cm^–1^ is associated with the out-of-plane vibrations of the semimetallic 1T′ phase. The intensity of the 1T′ peak is three times larger than that of the 2H phase, suggesting more 1T′ phase compared to 2H phase and indicating a partial phase transition.

Next, voltage-dependent Raman spectroscopy is performed to identify the voltage at which the phase transition occurs. The *V*_SG_ is varied from 3.5 to 0 to 3.5 V in the interval of 0.5 V. From 0 to 1.5 V, the positions of the characteristic 2H peaks are maintained, indicating no change in the semiconducting 2H phase as shown in Figure 3(d). For *V*_SG_ > 2 V, the intensity of the 2H peak at 171 cm^–1^ decreases and the 1T′ peak at 167.5 cm^–1^ emerges. A minimum *V*_SG_ = 2.5 V is required to see a distinct 1T′ phase peak; therefore, we regard 2.5 V as the phase transition voltage. Note that because *V*_SG_ ≫ *V*_DS_ in the voltage range where the phase transition is detected, the driving force for the phase change will primarily be provided by the *V*_SG_. Prior finite element modeling results show that applying a *V*_DS_ of 0.5 V decreases the ion concentration near the drain contact by less than 2%.^37^

The Raman data show that the intensities of both out-of-plane modes (171 (2H) and 167.5 (1T′) cm^–1^) are lower than the in-plane mode 233 (2H) cm^–1^ peak, which is expected because the out-of-plane vibrations are suppressed in a multilayer flake.^36^ Also note that the presence of the E_2g_ mode at 233 cm^–1^ and *V*_SG_ > 2.5 V indicates an incomplete phase transition; therefore, we classify it as a partial phase transition. Such coexistence of the 2H and 1T′ phases in Raman spectroscopy has been previously attributed to a layer-by-layer phase transition^12^ and the presence of both 2H and 1T′ domains under the same laser spot.^18^ In this work, the laser spot size is ∼1.4 μm, which is nearly three times larger than the 500 nm cavities. This means that some fraction of the signal will include a contribution from the supported, multilayer flake, making it unsurprising that both phases are detected.

Previous reports demonstrate that tensile strain induces redshifts in the in-plane phonon mode peak in TMDs.^38−40^ Here, a similar redshift in the E_2g_ peak is expected with an increase in the positive gate voltage. The position of the E_2g_ peak is shown on the left *y* axis in Figure 3(e) as a function of the applied gate voltage, where each peak value was determined by a Lorentzian fit. The shift in the peak position from the unstrained MoTe_2_ is reported as Δ Raman shift on the right *y* axis in Figure 3(e). The peak position is red-shifted by 0.75 cm^–1^ when the gate voltage is increased from 0 to 3 V. This observation confirms the existence of voltage-induced tensile strain in the suspended MoTe_2_ flake. Specifically, in the *V*_SG_ range where the onset of the partial phase transition is detected (2.5–3 V), the peak position is red-shifted by 0.3–0.6 cm^–1^, respectively. Note that although the E_2*g*_ mode is not sensitive to doping, high carrier densities may shift the peak position slightly to lower wavenumbers. To consider what fraction of the E_2g_ mode shift is due to doping versus straining, the E_2g_ mode peak positions of the supported MoTe_2_ FET are compared at *V*_SG_ = 0 and +3 V. No significant redshift is observed in either the E_2g_ or the A_1g_ peaks as reported in the Supporting Information Part 6.

**Figure 4:**
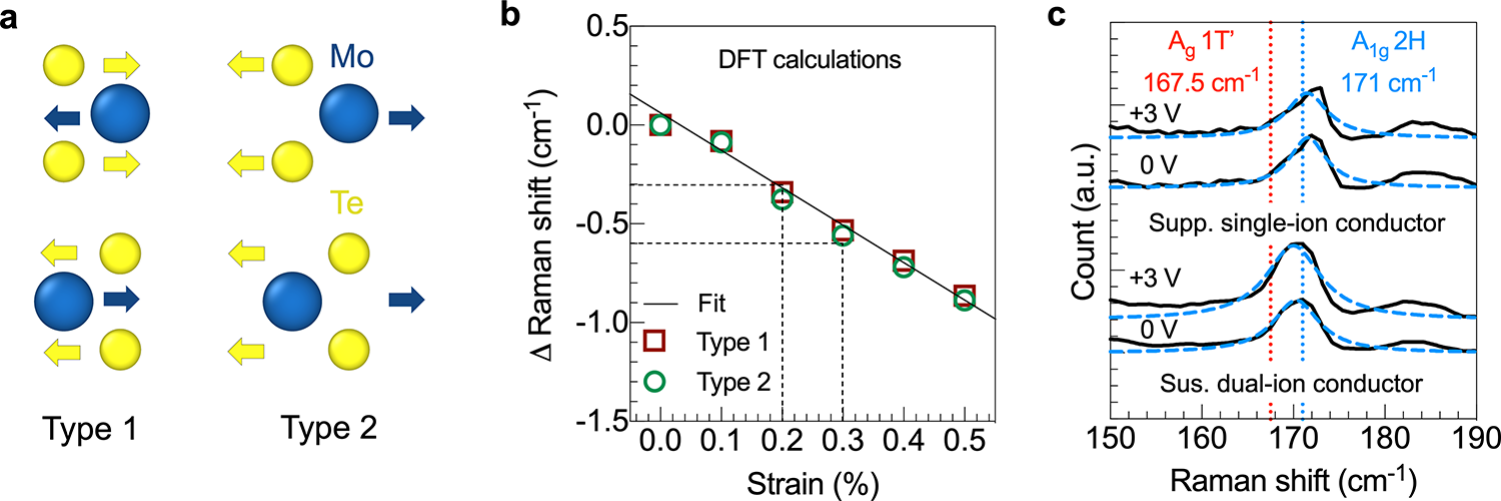
DFT calculation results and Raman control measurements: (a) Two types of atom movement in bilayer MoTe_2_ for DFT frequency calculations. The same atoms in two different layers move in the opposite (same) direction in type 1 (type 2). (b) Shift in the E_2g_ peak position relative to the unstrained position as a function of applied strain from the DFT calculations. (c) Raman spectra of the out-of-plane vibration mode in a supported MoTe_2_ FET gated using a single-ion conductor (top) and suspended MoTe_2_ FET gated using a dual-ion conductor (bottom) at *V*_SG_ = 0 and +3 V.

To quantify the induced strain based on the Raman peak shift, DFT calculations were performed by imparting known strain values to bilayer MoTe_2_ to calculate the shift in the Raman frequency (see Methods). Bilayer MoTe_2_ was isotropically strained at 0.1–3% and the phonon frequencies were calculated using a finite-difference method. Two types of in-plane modes were considered: in type 1, two layers were restricted to vibrating out-of-phase, whereas in type 2, layers were restricted to vibrating in-phase (Figure 4(a)). The Δ Raman shift values for strain in the range of 0 to 0.5% are reported in Figure 4(b). DFT calculations predicted an ∼1 cm^–1^ shift in the Raman peak with 0.5% applied strain. As a reminder, the experimentally measured Δ Raman shift at the phase transition voltage is 0.3–0.6 cm^–1^ (Figure 3e), corresponding to an estimated strain of ∼0.2–0.3% by DFT (Figure 4b). The estimated strain values are in agreement with the previously reported strain values for phase transition by experiments (0.2%)^18^ and simulations (0.3–3%).^11^

To further strengthen the claim of a strain-induced, partial phase transition, simultaneous electrical transport and Raman spectroscopy measurements were performed on two types of control devices that are not expected to undergo the transition: (1) suspended MoTe_2_ FETs gated using a dual-ion conductor and (2) supported MoTe_2_ FETs gated using the single-ion conductor. Under an applied bias, a dual-ion conductor, poly(ethylene oxide) lithium perchlorate (PEO:LiClO_4_), will create a symmetric EDL at the gate/electrolyte and the electrolyte/semiconductor interfaces, which should result in no net strain on MoTe_2_; therefore, no phase change is expected. Similarly, a supported FET gated using PE900-Li will generate stress but will not be able to strain the MoTe_2_ because it is supported by h-BN. Simultaneous electrical and Raman measurements on both control devices show no emergence of the 1T′ peak at *V*_SG_ = +3 V (Figure 4(c)). The absence of a phase transition in the control measurements for which no strain is expected further supports the voltage-induced tensile strain mechanism.

Prior theoretical and experimental reports have claimed that a large density of charge carriers (∼10^14^ cm^–2^) can induce the phase change;^16,41^ however, the mechanism has since been confirmed as an electrochemical one involving the formation of Te vacancies^12^ and not electrostatic doping alone. In this work, the side gate leakage current always remains less than ∼1 nA with no abrupt changes in current that are a signature of Faradaic contributions (Supporting Information Part 7). These observations support the conclusion that the phase transition is not mediated by electrochemical mechanisms. Even though establishing a high doping density cannot induce the phase transition, it likely reduces the energy difference between the 2H and 1T′ phase, which in turn decreases the strain required to induce the transition.^16^ We have experimentally measured the charge carrier density of a supported, MoTe_2_ FET gated by the single-ion conductor using the Hall effect as 2 × 10^13^ cm^–2^ at *V*_SG_ = 2 V.^33^ Thus, we expect this high doping density induced by ions to facilitate the transition at a lower strain than would otherwise be expected in an undoped system.

**Figure 5:**
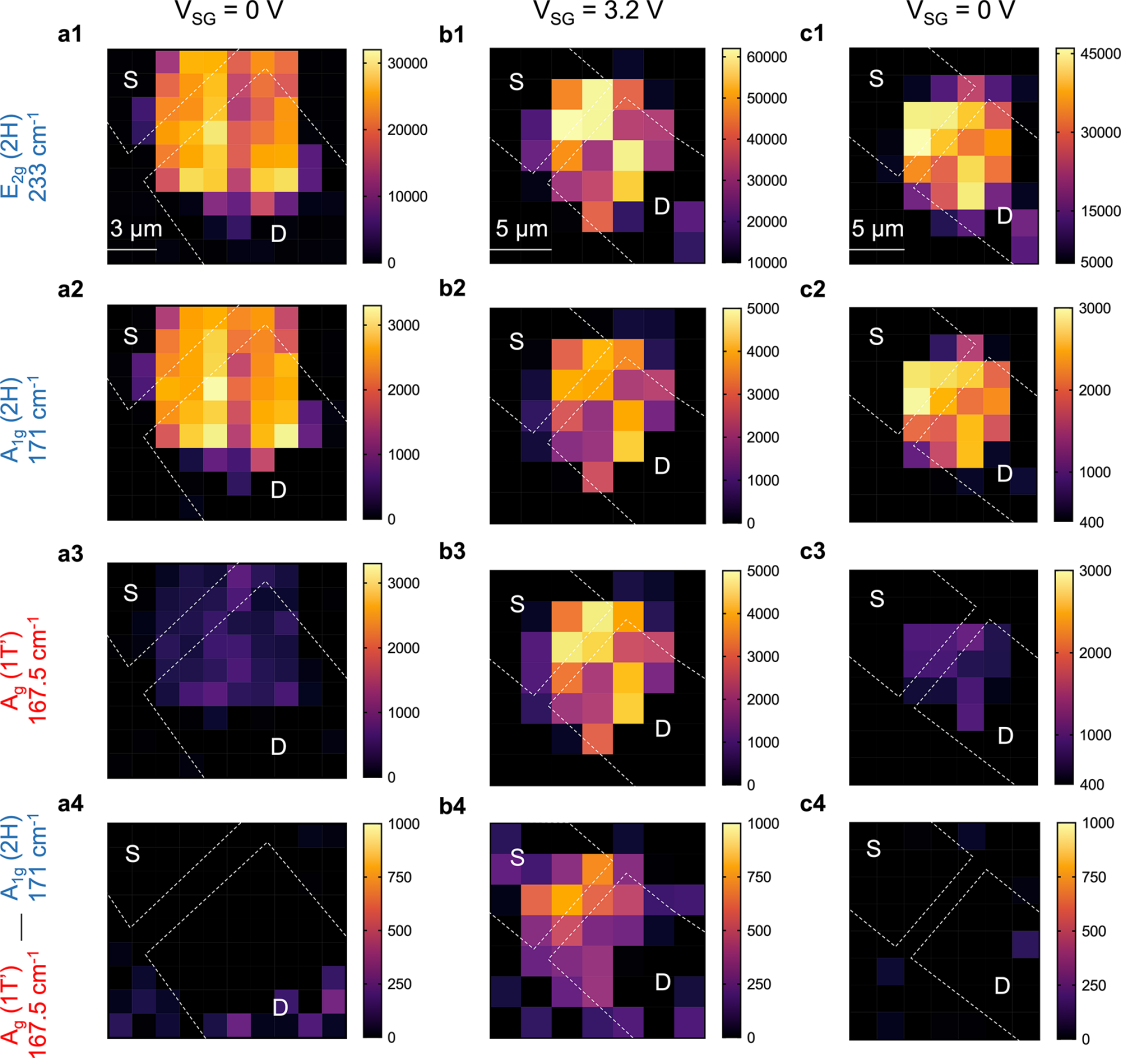
Raman intensity maps of the suspended MoTe_2_ (thickness ∼7.5 nm) device coated with a single-ion conductor. Characteristic 233 cm^–1^ (E_2g_ 2H), 171 cm^–1^ (A_1g_ 2H), and 167.5 cm^–1^ (A_g_ 1T′) peaks and the difference between A_g_ 1T′ and A_1g_ 2H, i.e., 167.5–171 cm^–1^ at (a) *V*_SG_ = 0 V, (b) *V*_SG_ = +3.2 V, and (c) *V*_SG_ = 0 V. Dashed lines highlight the border of the source/drain contacts. The region between the S/D contacts is suspended over 20 holes. The 1T′ peak appears at 3.2 V, indicating the phase transition, and disappears after removing the gate voltage, indicating reversibility. Note that the color bar ranges for 171 and 167.5 cm^–1^ peaks are set to be equivalent for a direct comparison between the two maps. Each pixel equals 1.5 μm in column a and 2.5 μm in columns b and c.

To study the spatial distribution of the phase transition, Raman mapping was performed on the entire device. Focusing on the key Raman peaks at 167.5 (1T′), 171 (2H), and 233 (2H) cm^–1^, the flake is mapped in the absence of a gate voltage (Figure 5(a)). The intrinsic 2H phase is confirmed by strong intensities at 233 and 171 cm^–1^ across the entire flake, as expected for *V*_SG_ = 0 V (Figure 5(a1 and a2)). Moreover, the intensity of the 167.5 cm^–1^ (1T′) peak is close to zero across the entire flake (Figure 5(a3)). This observation confirms that gravity alone does not induce a 1T′ phase.

As shown in Figure 5(b), when the FET is biased to *V*_SG_ = 3.2 V, a Raman signal emerges at 167.5 cm^–1^, indicating the presence of the 1T′ phase. Note, however, that the peaks at 171 and 233 cm^–1^ remain, meaning that the 2H phase also persists to some extent at *V*_SG_ = 3.2 V as previously mentioned in Figure 3(d). From the mapping, we learned that the 1T′ phase signal is present across the entire area of the flake, including the supported regions.

Some details of the spatial distribution can be identified by plotting the difference between the intensities of 1T′ and 2H phases, i.e., 167.5–171 cm^–1^, which we will refer to as a difference map. As expected, Figure 5(a4) and (c4) contain no regions with higher 1T′ intensity at *V*_SG_ = 0 V because the 1T′ phase has not been induced. At *V*_SG_ = 3.2 V (Figure 5 b4), the difference map highlights the presence of the 1T′ phase while revealing an important detail not apparent in the first three rows of maps: the intensities associated with the 1T′ phase are stronger than the 2H phase in the suspended region specifically (i.e., the region between the S/D contact, see Supporting Information Part 8 for an overlay of the difference map and AFM topology image). When the suspended region is moved away, the intensity decreases, corresponding to an increasing 2H phase signal. A similar observation is made on device 2 with two 500 nm cavities (see Supporting Information Parts 9 and 10 for characterization and Raman maps of device 2).

After the gate bias is removed, the intensities corresponding to the 1T′ phase disappear (Figure 5(c)), and only the 2H phase peaks remain, showing that the partial phase transition is reversible. Additional phase switching was performed by alternatively applying 3 and 0 V to the side gate to repeatedly check for any retention of the 1T′ phase. No evidence of 1T′ phase retention was found after six consecutive 2H–1T′ phase transitions (Supporting Information, Part 11). These results further demonstrate the reversibility of the phase switching, and the fully reversibly nature agrees with the strain-based mechanism. Note that the Raman mapping reported in Figure 5 is performed over a time scale of a few hours. Prolonged exposure to the high-intensity laser is reported to induce a permanent phase transition in 2H-MoTe_2_.^15^ To eliminate the possibility of a heat-induced phase transition during mapping, Raman spectra were acquired before and after the mapping; no evidence of a permanent phase transition was found (Supporting Information, Part 12).

To study the dynamics of the phase change, time-dependent Raman spectroscopy is performed. The difference between the intensities of the out-of-plane vibration modes of the 1T′ and 2H phase is monitored as a function of time (shown in Supporting Information Part 13). Both the 1T′ to 2H and 2H to 1T′ transitions occur over a time scale of ∼200 s. Duerloo et al.^11^ and Berry et al.^42^ predicted the phase transition time scales by DFT calculations on the order of ∼50 s and ∼10 min, respectively. Experimentally Song et al.^18^ reported a phase transition time of ∼40 min. In our case, the phase transition time is a combination of ion response time (i.e., EDL formation and dissipation time) and the intrinsic phase transition time of the MoTe_2_. Note that the EDL formation time is ∼20 s (see Supporting Information Part 14), which is significantly smaller than the observed phase transition time of ∼200 s. To ensure that the phase change dynamics with voltage are unaffected by the acquisition time of the Raman measurements, gate voltages were applied for 15 min for all *V*_SG_ prior to collecting Raman spectra. This time scale is five times longer than the data acquisition time itself, ensuring that the voltage-dependent response is induced prior to the Raman measurement.

**Figure 6:**
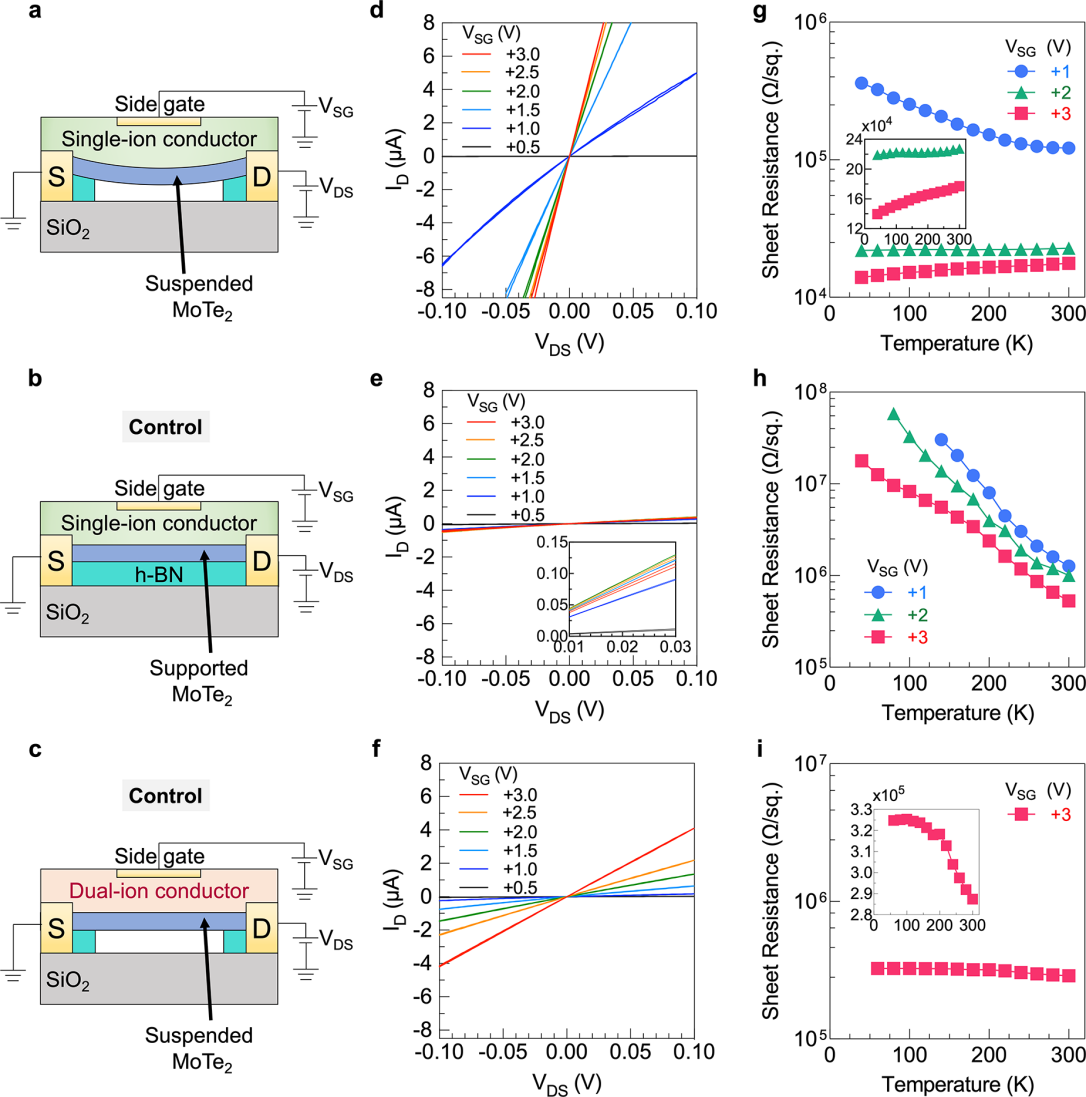
Electrical characterization: Schematic of the (a) suspended and (b) supported MoTe_2_ FET gated using a single-ion conductor. (c) Schematic of the suspended MoTe_2_ FET gated using a dual-ion conductor. (d) Output characteristics (*I*_D_–*V*_DS_) of the device represented in (a). A sudden change in the *I*_D_–*V*_DS_ slope is observed for *V*_SG_ > 1.5 V, attributed to the 2H to 1T′ partial phase transition. *I*_D_–*V*_DS_ (e and f) of the control devices represented in (b) and (c), respectively. The insets represent the zoomed-in *x*-axis from 10 to 30 mV. Temperature-dependent sheet resistance of the (g) suspended MoTe_2_ FET gated using a single-ion conductor, (h) supported MoTe_2_ FET gated using a single-ion conductor, and (i) suspended MoTe_2_ FET gated using a dual-ion conductor. Positive slopes at *V*_SG_ = 2 and 3 V in (g) indicate metallic-type conduction, confirming the existence of the 1T′ phase. Negative slopes at *V*_SG_ = 1 V in (g) and all gate voltages in (h) and (i) indicate semiconducting-type conduction, confirming the 2H phase. The insets show linear scale sheet resistance.

While Raman spectroscopy provides a spectroscopic confirmation, the transition is also confirmed electrically by measuring the output characteristics and temperature-dependent resistance of a suspended MoTe_2_ FET gated with the single-ion conductor (Figure 6(a)) and control devices. For the suspended MoTe_2_ FET gated using a single-ion conductor, the output characteristics show ohmic behavior, i.e., a linear increase in *I*_D_ with *V*_DS_, as expected (Figure 6(d)). However, the slope of *I*_D_ vs *V*_DS_ changes significantly for *V*_SG_ > +1.5 V, indicating a sharp increase in the channel conductance, consistent with a phase change. Note that the current compliance is set to ∼8 μA to protect the channel from carrying an excessive current. The channel conductance increases from 0.13 μA/V to 290 μA/V (∼2200× increase) when *V*_SG_ increases from +0.5 V to +3.0 V. In addition, the channel resistance as a function of *V*_SG_ and the transfer characteristics of the suspended MoTe_2_ FET (device 1) are reported in Supporting Information Part 7.

Focusing now on the control devices, for the same increase in *V*_SG_, the conductance of the supported MoTe_2_ FET gated using a single-ion conductor increases by only ∼8.5 times (Figure 6(e)) and that of the suspended MoTe_2_ FET gated using a dual-ion conductor by only ∼130 times (Figure 6(f)). Moreover, no sudden change in slope is observed. Thus, the abrupt change in slope of the single-ion gated, suspended device can be attributed to the 2H to 1T′ phase transition in the voltage range of *V*_SG_ ≈ +1.5 to 2 V. Note that this voltage range is slightly smaller than the voltage range detected by Raman spectroscopy (∼2.5 V as shown in Figure 3(d)); however, both the electrically and spectroscopically detected voltage ranges in this study are ∼0.5–1 V smaller than the experimentally reported voltage induced by electrochemical doping using an ionic liquid (∼3–3.5 V for bilayer MoTe_2_).^12^

Although the abrupt change in the conductance can result from the semiconducting-to-semimetallic phase change, this evidence alone does not prove metallic-type conduction. Thus, temperature-dependent resistance measurements are made. With increasing temperature, the resistance of semiconductors decreases because of easier excitation of carriers across the gap;^43−45^ that is, semiconductors have a negative temperature coefficient of resistance (α). In contrast, the resistance of metals increases with increasing temperature because of increased molecular vibrations;^46−48^ that is, metals have positive α. Such contrasting behavior is expected in the semiconducting 2H phase MoTe_2_ versus the semimetallic 1T′ phase.

To measure the temperature-dependent resistance, *V*_SG_ was applied in the range of 1–3 V and held constant for 15 min to ensure EDL equilibration at 300 K. The temperature was then decreased to 40 K in 20 K intervals, and *I*_D_ was measured for 2 min at each temperature interval. Sheet resistance is reported for *V*_SG_ = 1, 2, and 3 V for the suspended MoTe_2_ FET gated using a single-ion conductor and the controls in Figure 6(g), (h), and (i), respectively.

For the suspended MoTe_2_ FET gated using a single-ion conductor, the sheet resistance increases from 121 kΩ/sq. to 361 kΩ/sq. at *V*_SG_ = 1 V when the temperature decreases from 300 K to 40 K (Figure 6(g)). The increasing resistance with decreasing temperature corresponds to a negative α of −2.55 × 10^–3^ K^–1^, proving the semiconducting character of the channel. However, when *V*_SG_ is increased to 3 V, the sheet resistance *decreases* from 17.6 kΩ/sq. to 13.9 kΩ/sq. over 300 to 40 K, corresponding to a positive α of +1.03 × 10^–3^ K^–1^. The slope at *V*_SG_ = 2 V is close to zero (α = +1.40 × 10^–4^ K^–1^), representing an intermediate state as the phase change progresses. These data electrically confirm the semiconducting-type conduction at *V*_SG_ = 1 V and metallic-type conduction at 3 V, with the onset around *V*_SG_ = 2 V, which is in agreement with the spectroscopically detected phase transition voltage presented above (∼2.5 V).

In contrast to the suspended device, the supported MoTe_2_ FET gated using a single-ion conductor shows purely semiconducting character (Figure 6(h)) with α = −5.99, −4.47, and −3.37 × 10^–3^ K^–1^ at *V*_SG_ = 1, 2, and 3 V, respectively. Similarly, the suspended MoTe_2_ FET gated using a dual-ion conductor also shows semiconducting behavior with α = −4.79 × 10^–4^ K^–1^ at *V*_SG_ = 3 V (Figure 6(i)). Thus, only the suspended device gated by the single-ion conductor undergoes the 2H to 1T′ transition.

## Conclusions

An approach is demonstrated to apply field-induced strain to transform a semiconducting 2H phase MoTe_2_ FET to the semimetallic 1T′ phase. Strain is applied by EDL gating using a single-ion conductor, enabling individual devices to be addressed and, therefore, strained electrically. A characteristic out-of-plane vibration mode (A_g_) for the semimetallic 1T′ phase is observed during simultaneous electrical and Raman spectroscopic measurements for *V*_SG_ > 2.5 V, confirming a phase transition. A redshift in the in-plane vibration mode (E_2g_) confirms tensile strain estimated by DFT calculations to be ∼0.2–0.3% at *V*_SG_ = 2.5–3 V. These values agree with those reported previously for MoTe_2_. The 1T′ phase is present across the entire area of the flake, and it reverts back to 2H when *V*_SG_ is removed, demonstrating the phase transition to be completely reversible.

The phase change is also confirmed electrically. A large change in slope is observed in the output characteristics at *V*_SG_ > 1.5 V corresponding to a sheet resistance that is 1 order of magnitude lower than the controls at the same *V*_SG_. The sheet resistance decreases with decreasing temperature from 300 K to 40 K (positive α) for *V*_SG_ = +2 and +3 V, which is a characteristic of metallic-type conduction through the 1T′ phase. In contrast, for the suspended device at *V*_SG_ = 1 V and in both control devices at all applied gate voltages, an increase in resistance with decreasing temperature (negative α) was observed, confirming the semiconducting 2H phase MoTe_2_. The approach presented here can be used to achieve location-specific, field-induced strain that induces a reversible phase transition using low voltage and can be extended to control the electrical properties of other types of 2D materials. Moreover, it provides a route to characterize strain-induced physical phenomena of low-dimensional materials and could be valuable for applications such as low-power phase-change memory and logic.

## Methods

### Suspended FET Fabrication

Few-layer h-BN was mechanically exfoliated onto 90 nm SiO_2_/p-type Si (Graphene Supermarket, resistivity 0.001–0.005 ohm·cm) using the Scotch tape method. h-BN flakes of uniform thickness (50–60 nm) were selected by optical microscopy and AFM (Bruker Dimension Icon). Source and drain contacts were patterned using e-beam lithography (EBL; Raith e-LINE) with PMMA-950-A4 resist (MicroChem) at 4000 rpm for 1 min. The pattern was developed in a methyl isobutyl ketone (MIBK)/IPA solution (1:3 by volume) for 1 min and rinsed in IPA for 1 min. h-BN was etched using 30 sccm SF_6_ and 10 sccm O_2_ plasma with inductively coupled plasma–reactive ion etching (ICP-RIE; PlasmaTherm APEX). Ti/Au contacts (5/55 nm) were deposited by e-beam evaporation (Plassys electron beam evaporator MEB550S). The metal thickness was set to equal the depth of the etched h-BN such that the surface of the metal and the h-BN lie in the same plane. The lift-off was performed by soaking the samples in acetone for 24 h. Cavities in the h-BN between S/D electrodes are patterned by EBL using the same method as described above. Cavity depth was measured by AFM (Figure 2b).

Few-layer MoTe_2_ (2D Semiconductors) was exfoliated and identified the same as h-BN. The exfoliated flakes of thickness 5–7 nm were picked up by a PC/PDMS stamp and aligned over the cavities using an optical microscope. The stamp was pressed onto the substrate using micromanipulator movement in the vertical direction, making sure the flake was in contact with the source/drain metal. The structure was heated to 185 °C to release the PC and flake on the substrate. The remaining PC was removed by dissolving it in chloroform. AFM was used to confirm the suspension of the flake in the cavity. Output and transfer measurements of the suspended MoTe_2_ FET using the SiO_2_ back gate are reported in Supporting Information Part 15.

### Single-Ion Conductor Deposition

A 3 wt % solution of the single-ion conductor (PE900-Li) in DMF was prepared inside an Ar-filled glovebox. The synthesis details are described by Dou et al.^32^ The solution was drop-cast onto MoTe_2_ FETs (50 μL on 1 cm^2^ SiO_2_/Si). DMF evaporated naturally inside the glovebox overnight. The samples were transferred to the probe station for electrical measurements using the Ar-filled load lock with no exposure to ambient.

### Electrical Measurements

Electrical measurements were made using a Lakeshore cryogenic probe station with a vertical field superconducting magnet (CRX-VF) and using a Keysight B1500A semiconductor parameter analyzer. The temperature of the sample stage was maintained at 300 K under ∼5 × 10^–7^ Torr. For output measurements, *V*_SG_ was held constant for 900 s to provide sufficient time for the ions to reach a steady state before starting the measurement. Then, *V*_DS_ was swept at 4 mV/s and the *I*_D_ was monitored. The same sequence was repeated for all of the *V*_SG_ and control measurements. For the temperature-dependent resistance measurements, *V*_SG_ was held constant for 900 s at 300 K before the temperature was decreased at ∼1 K/min in 20 K intervals while monitoring *I*_D_.

### Raman Spectroscopy Measurements

Raman spectroscopy was performed while making electrical measurements using a custom-built setup depicted in Figure 3(a). The single-ion conductor absorbs water in ambient conditions; therefore, N_2_ gas was flowed over the device during the measurements to minimize air exposure. Three portable electrical measurement probes were attached to the micromanipulators (Sinatone S-725), and two SMUs (Keithley 2450) were used to apply *V*_DS_ and *V*_SG_. *V*_SG_ was applied and held constant for 900 s before the Raman spectral acquisitions. A Renishaw inVia Raman spectrometer with a laser excitation wavelength of 633 nm was used to acquire the spectra (10 s each) in the dark; the reported spectra are an accumulation of three consecutive measurements. The spot diameter of the Raman laser is estimated as 1.22 × λ/NA = 1.4 μm, where λ is the wavelength (633 nm) of the light used and NA is the numerical aperture (0.55) of the 50× objective lens. The laser power density was kept under ∼1 mW/μm^2^ to limit the local heat induced by laser irradiation. Note that there is at least a micrometer thick polymer on top of the MoTe_2_, and therefore, the actual power density reaching the MoTe_2_ will be lower than 1 mW/μm^2^ and does not cause significant heating, as supported by the Raman study in the Supporting Information Part 12.

### DFT Calculations

Raman frequencies were simulated in VASP^49−51^ using a PBE functional and PAW pseudopotentials.^52^ A bilayer MoTe_2_ system (initial coordinates taken from the Materials Project database^53^) was relaxed to eliminate any remnant strain in the simulation conditions (9 × 9 × 1 gamma-centered *k*-points, 820 eV plane-wave energy cutoff, spin-orbit coupling included, DFT-D3(zero) van der Waals corrections included^54^). The system was isotropically strained through an affine transformation on the atoms and unit cell to a series of coarse steps in strain percentage (around 0.3% each up to 3.0% strain) and a similar series of fine steps (0.1% strain up to 1.2%). In each of these systems, the atoms were then re-relaxed with the unit cell parameters fixed, and the phonon frequencies were found using a finite-difference method. This produces more than one *E*_2g_-type motion, as the independent layers can vibrate in different directions with only a small effect on the mutual frequency. In this case, the two modes with both layers oscillating completely in-phase and completely out-of-phase were used.

## Supporting Information Available

Device fabrication schematic; MoTe_2_ flake surface characterization; Raman characterization of the 2H-MoTe_2_ flake, single-ion conductor, E_2g_ mode; electrical measurements of the suspended flake; optical images of suspended FETs under Raman microscope; difference maps overlaid on AFM scans; fabrication and characterization of the suspended MoTe_2_ FET (device 2); Raman mapping (device 2); demonstration of reversibility by gate voltage switching; Raman spectra of suspended MoTe_2_ FET before and after mapping; dynamics of phase transition; dynamics of EDL formation; electrical characterization of bare FET (PDF)
