# Supplementary material for: Strain-Induced 2H to 1T′ Phase Transition in Suspended MoTe2 Using Electric Double Layer Gating
Source: ACS Nano. 2023 Nov 10;17(22):22388–98. doi: 10.1021/acsnano.3c04701 (PMC10690768; doi:10.1021/acsnano.3c04701)
Supplement: Supplementary file 1 — nn3c04701_si_001.pdf [file nn3c04701_si_001.pdf]

Supplementary Information - Strain-induced 2H to 1T′ Phase Transition in Suspended $MoTe_2$ using Electric Double Layer Gating

Shubham Sukumar Awate,[†] Ke Xu,[‡,¶,†] Jierui Liang,[†] Benjamin Katz,[§] Ryan Muzzio,[‖] Vincent H. Crespi,[⊥,§,⊥,#] Jyoti Katoch,[‖] and Susan K. Fullerton-Shirey[*,†,@,△]

†*Department of Chemical and Petroleum Engineering, University of Pittsburgh, Pittsburgh, Pennsylvania 15260, United States*

‡*School of Physics and Astronomy, Rochester Institute of Technology, Rochester, New York 14623, United States*

¶*Microsystems Engineering, Rochester Institute of Technology, Rochester, New York 14623, United States*

§*Department of Physics, The Pennsylvania State University, University Park, Pennsylvania 16802, United States*

‖*Department of Physics, Carnegie Mellon University, Pittsburgh, Pennsylvania Department of Physics, Carnegie Mellon University, Pittsburgh, 15213, United States*

⊥*Department of Materials Science and Engineering, The Pennsylvania State University, University Park, Pennsylvania 16802, United States*

#*Department of Chemistry, The Pennsylvania State University, University Park, Pennsylvania 16802, United States*

@*Department of Electrical and Computer Engineering, University of Pittsburgh, Pittsburgh, Pennsylvania 15260, United States*

△*Current address: 3700 O'Hara Street, Pittsburgh, Pennsylvania 15213, United States*

E-mail: fullerton@pitt.edu

1. Schematic of the suspended $MoTe_2$ FET fabrication process

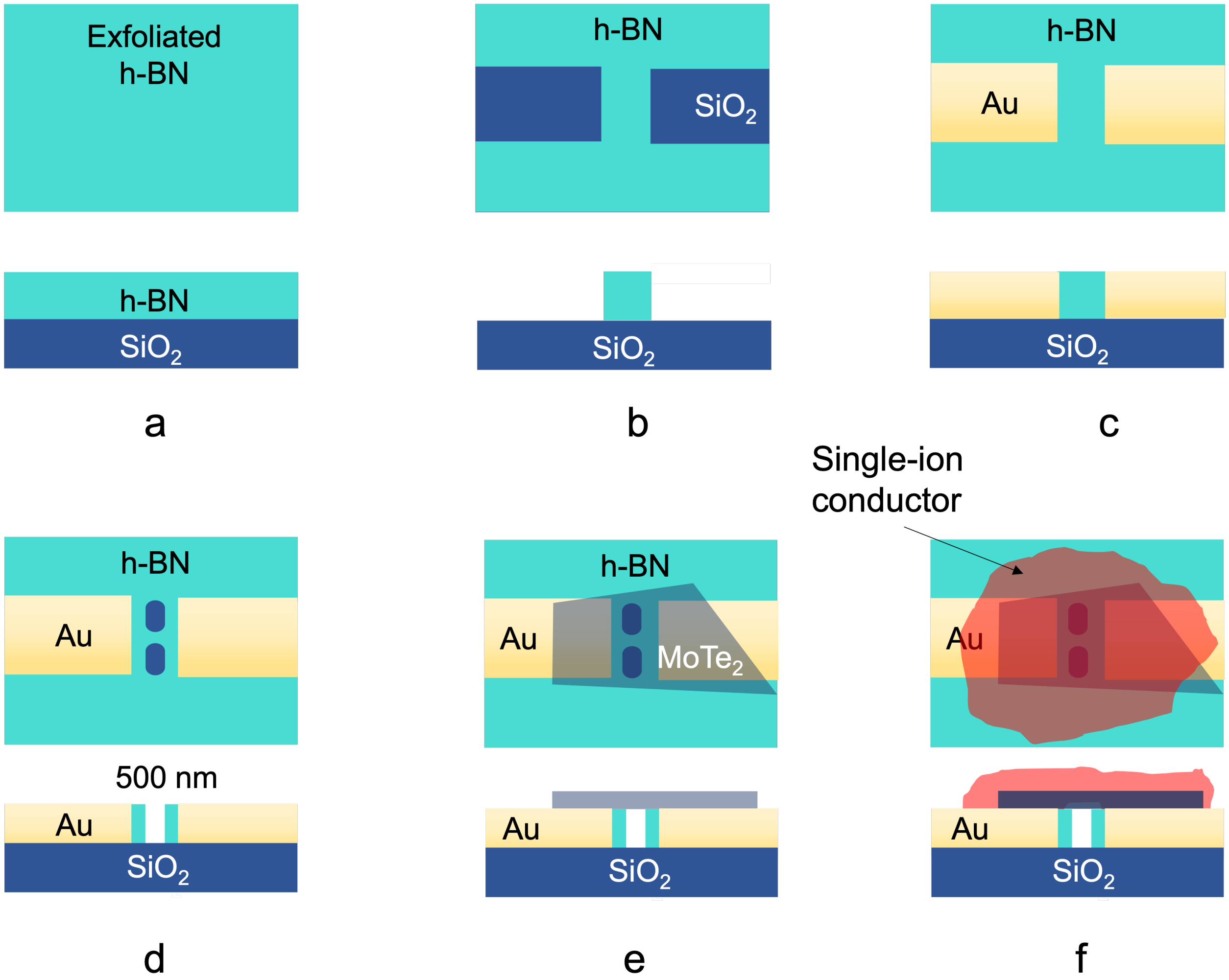

Figure S1: Top and cross-sectional views of the suspended $MoTe_2$ FET (a) exfoliation, (b) e-beam lithography followed by plasma etching, (c) metal evaporation with metal thickness equal to the h-BN thickness, (d) e-beam lithography followed by plasma etching to create the suspension area holes, (e) dry-flake transfer using PC/PMMA stamp, (f) deposition of single-ion conductor by drop-casting.

2. $MoTe_2$ Surface characterization (Device 1)

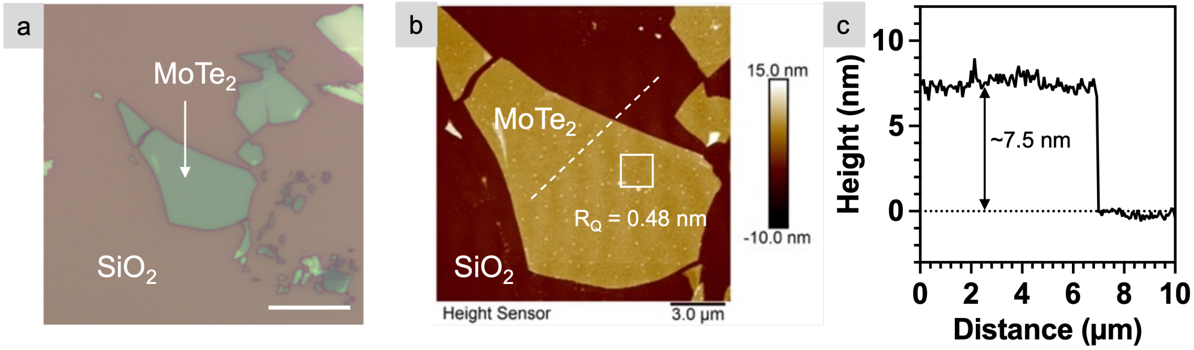

Figure S2: (a) Optical image of the $MoTe_2$ flake prior to device fabrication. The scale bar is 10 μm. (b) AFM topography scan of the same flake as (a). The root mean square roughness (R_q) is an average of three, 2×2 μm AFM scans at different locations on the flake. (c) Line scan of the AFM height image, corresponding to the dashed white in in (b)). The height of the flake is ~7.5 nm (~9−10 layers).

3. Complete Raman spectrum of the 2H $MoTe_2$ flake

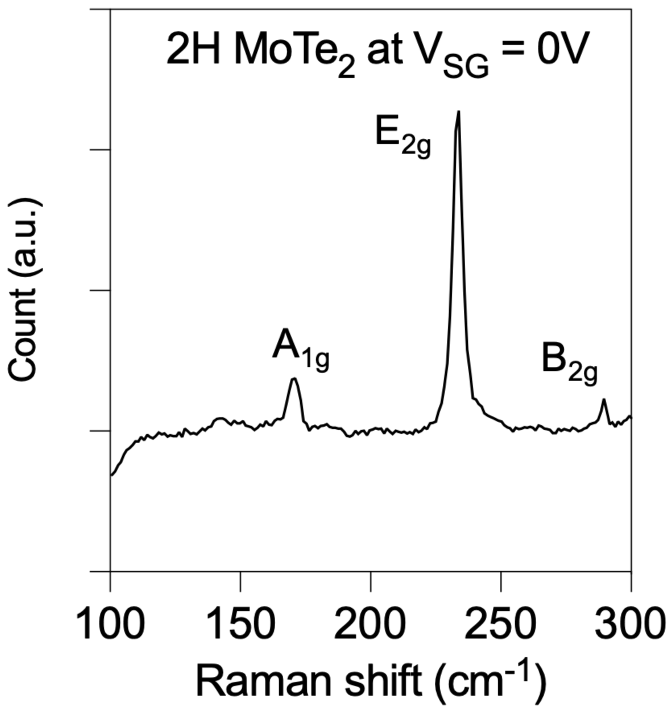

Figure S3: Raman spectra of the supported $MoTe_2$ device without the single-ion conductor.

4. Raman characterization of the single-ion conductor

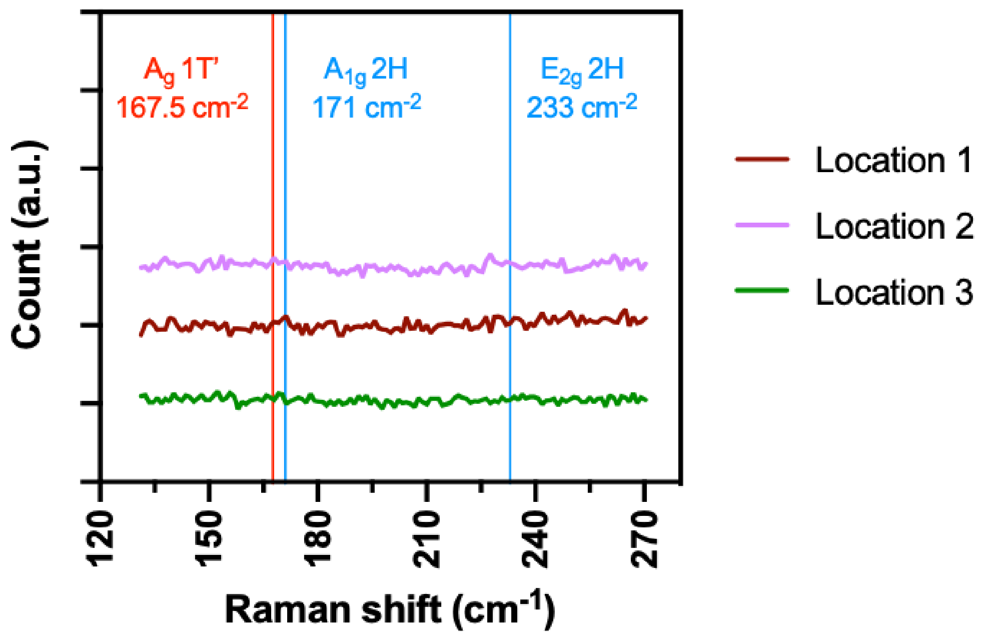

Figure S4: Raman spectra of the single-ion conductor excited using 633 nm wavelength at three different locations on SiO_2. There are no Raman active modes in the range of $130 - 270$ cm^{-1}, which means there is no contribution to the $MoTe_2$ spectrum from the single-ion conductor.

5. E_{2g} mode Raman peak of the supported and suspended $MoTe_2$ FET

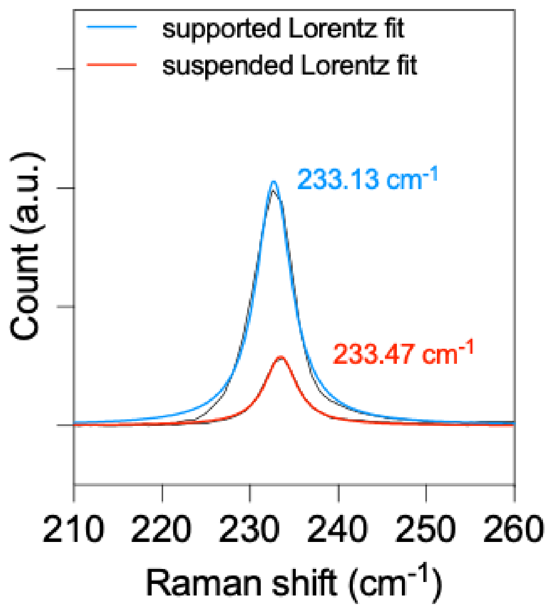

Figure S5: E_{2g} mode Raman peak of the supported and suspended MoTe2 FETs at V_{SG} = 0 V. Negligible difference in the peak position confirms that there is no significant built-in strain because of $MoTe_2$ sagging into the cavity

6. Raman spectra of supported $MoTe_2$ FET gated using single-ion conductor

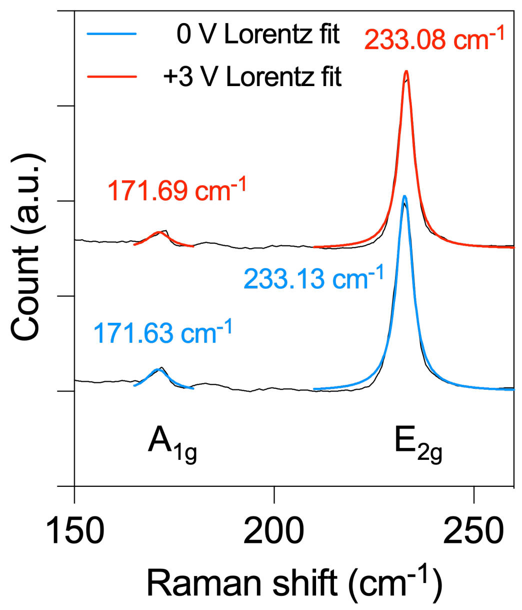

Figure S6: E_{2g} and A_{1g} mode Raman peaks of the supported MoTe2 FET gated using single ion conductor at $V_{SG} = 0$ and $+3$ V. No significant shift in the E_{2g} peak is observed compared to the shift reported for suspended $MoTe_2$ FET.

7. Electrical measurements as a function of V_{SG} for the suspended $MoTe_2$ FET (Device 1)

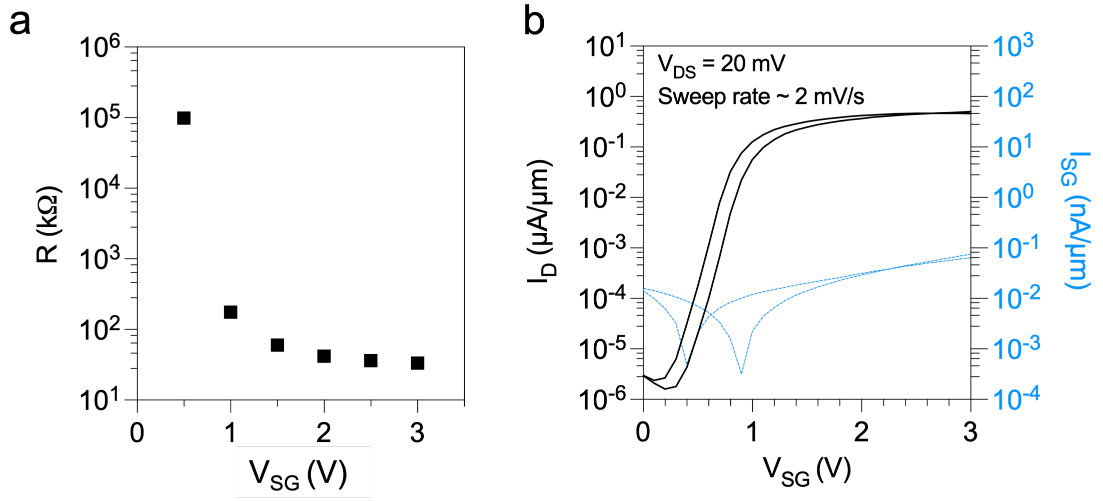

Figure S7: Electrical measurements as a function of gate voltage for device 1 (suspended MoTe2 with single-ion conducting gate) (a) Resistance extracted from the output measurements reported in Figure 6. (b) Transfer characteristics and gate leakage current, (I_{SG}). No indication of electrochemical reaction was detected in the I_{SG}.

8. Difference map overlaid on AFM scan

As mentioned in the main text, the difference map reveals that the intensities of the 1T′ are stronger in the suspended region indicating that the phase transition happens mainly near the suspended region. In supplementary figure S8), AFM topography scans for devices 1 and 2 are overlaid with the difference map to strengthen the claim of location-specific strain.

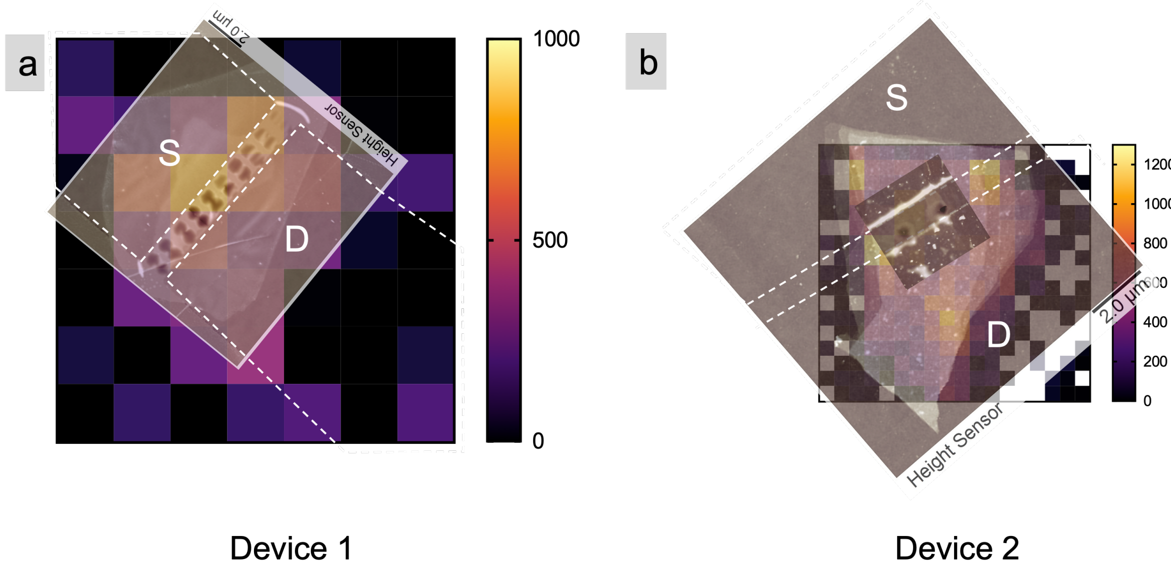

Figure S8: The difference between out-of-phase Raman vibration intensities of 1T′ and 2H phase overlaid with AFM topology scans for (a) device 1 and (b) device 2. The maps are generated by subtracting the signal intensities of the 2H phase from the 1T′ phase at every pixel, which are taken from the same spectral acquisition. Baseline correction was performed before subtracting the intensities to make the difference more accurate.

9. Fabrication and characterization of the suspended $MoTe_2$ FET (Device 2)

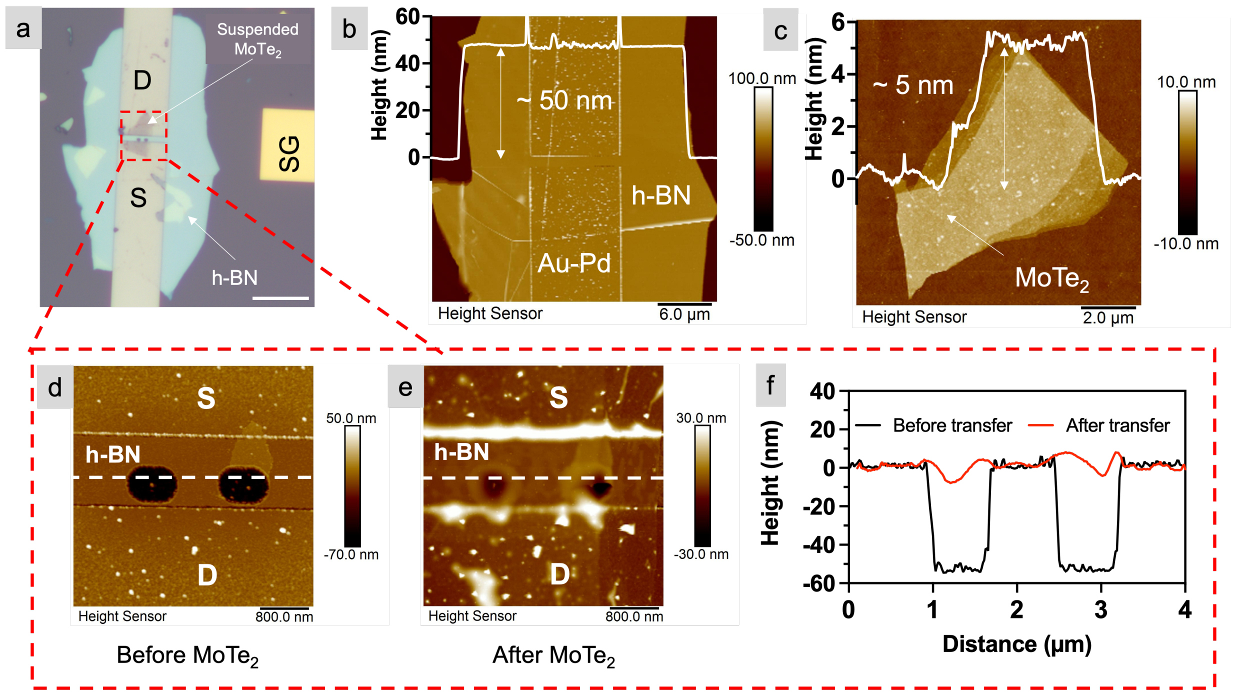

Figure S9: (a) Optical image of the fabricated device (scale bar = 10 μm) and AFM topography scans of the (b) h-BN substrate and (c) $MoTe_2$ flake. The S/D contacts are evaporated to the equivalent height of the h-BN flake (∼50 nm). The height of $MoTe_2$ flake is ∼ 5 nm (∼6−7 layers). AFM line scans over the full width of the topography plot are overlapped on the plot. AFM topography scan of two stadium-shaped trenches etched between the S and D contacts (d) before and (e) after the $MoTe_2$ flake transfer. (f) Trench depth before and after the $MoTe_2$ transfer; the depth after transfer is 5 times smaller than before which proves that the flake is suspended over both the trenches. Note that the appearance of sagging in the AFM image is exaggerated because the length scale of the X axis is ∼1000× larger than Y axis.

10. Raman mapping device 2

Measurement reported in Figure 5 for device 1 are repeated here on device 2.

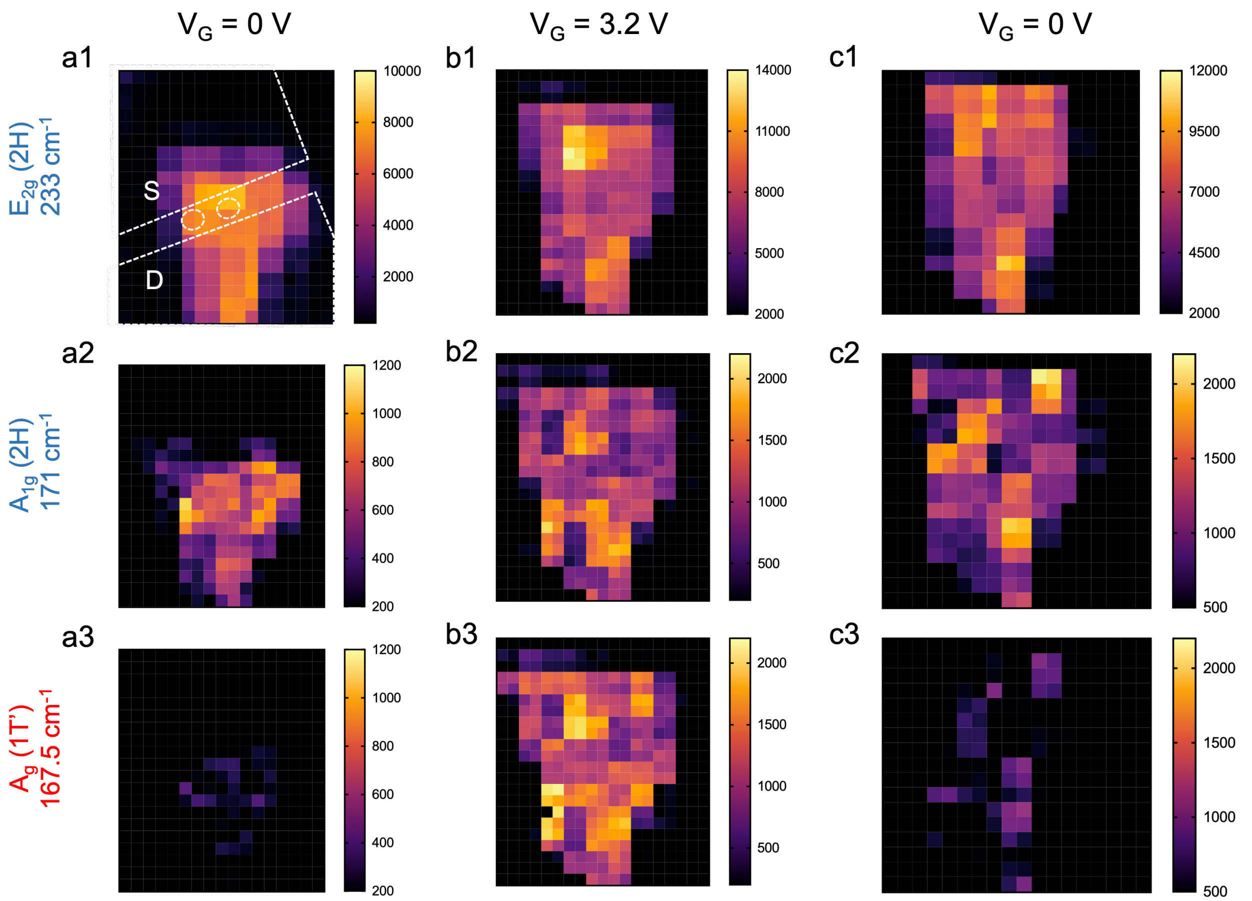

Figure S10: Raman maps of the suspended $MoTe_2$ device 2 (thickness ~5 nm) with ~ 1 μm film of single-ion conductor. Each column represents a characteristic peak: (1) 233 cm^{-1} (E_{2g} 2H), (2) 171 cm^{-1} (A_{1g} 2H) and (3) 176.5 cm^{-1} (A_{1g} 1T′) at (a) $V_G = 0$ V, (b) $V_G = +3.2$ V and (c) $V_G = 0$ V. Note that the color bar range for the 171 and 167.5 cm^{-1} peaks are set to be equivalent for a direct comparison between the two maps.

11. Reversible switching

To demonstrate repeated switching between the phases, the gate voltage was switched between $V_{SG} = 3$ V and 0 V and Raman spectra were acquired. No evidence of 1T′ phase retention was found at $V_{SG} = 0$ V during six consecutive 2H−1T′ phase transitions (Shown in Supplementary Figure S11).

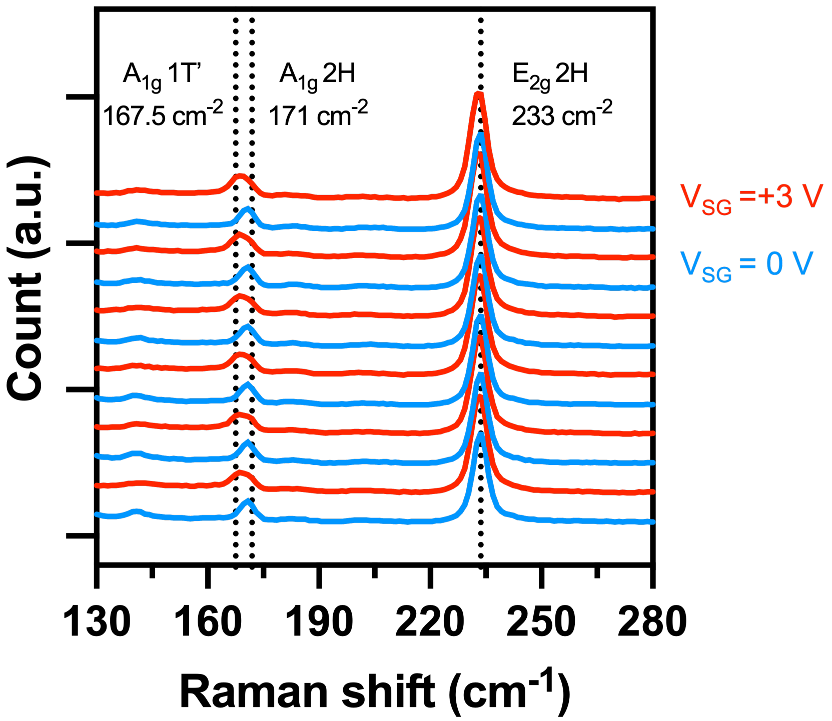

Figure S11: Raman spectra of suspended $MoTe_2$ FET after repeatedly applying 3 V to the side gate (red spectra) and removing the gate voltage (blue spectra). No evidence of 1T′ was retained after removing the gate voltage suggesting that the phase transition is fully reversible.

12. Raman spectra of suspended $MoTe_2$ FET before and after mapping

Raman maps reported in the manuscript are collections of three spectral acquisitions (each of 10 s) at every pixel in the map. The mapping exposes the $MoTe_2$ flake to a high intensity laser (power density $<\sim 1$ mW/μm^2) for few hours which can induce a permanent phase transition in 2H-$MoTe_2$.[1] The possibility that the phase transition occurs due to exposure to the laser is disproven by the Raman spectra below that show no change before and after mapping.

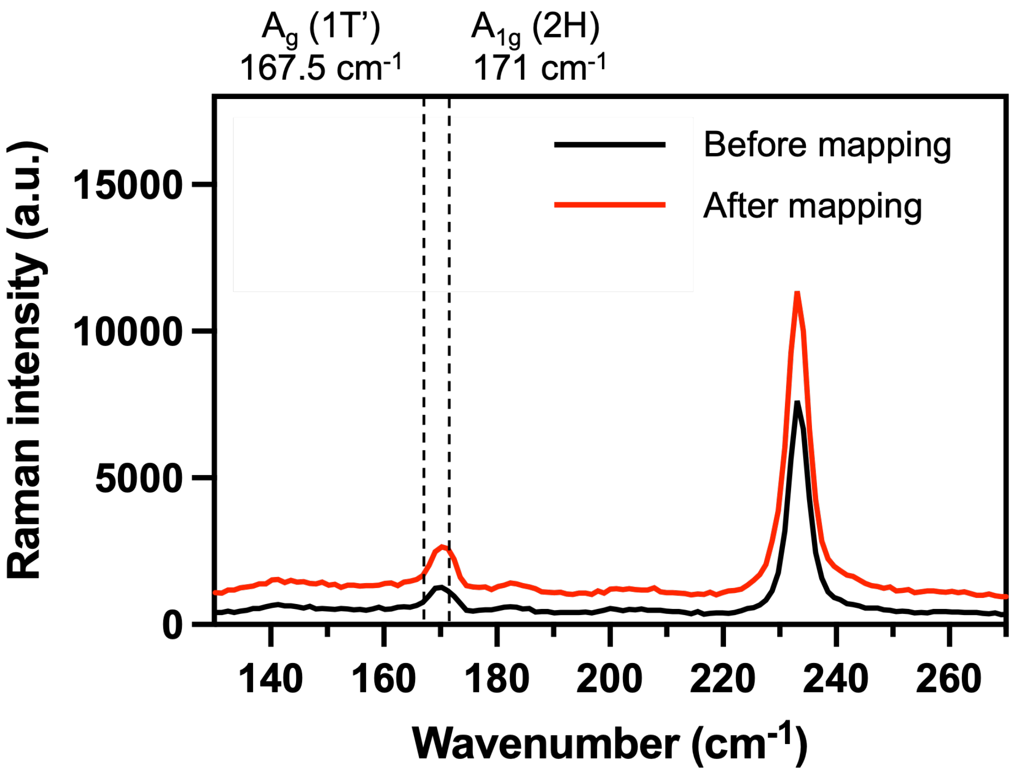

Figure S12: Raman spectra of a suspended $MoTe_2$ FET (device 2) with ~ 1 μm film of the single-ion conductor before and after the mapping.

13. Phase transition dynamics

Time-dependent Raman spectroscopy is performed to calculate the time required for the phase transition to be detected. A gate voltage of +3 V is applied to an intrinsic 2H phase $MoTe_2$ device and Raman spectra were acquired (each spectrum for 30 s) in a continuous mode for 500 s at three different locations on the flake. Time-dependent difference between the intensities of out-of-plane vibration mode of 1T′ and 2H phase is reported in Supplementary figure S13). Positive values on the Y axis represent the 1T′ phase whereas negative values represent 2H phase. It takes $\sim$ 200 s for both the 1T′ $\rightarrow$ 2H and 2H $\rightarrow$ 1T′ phase transition.

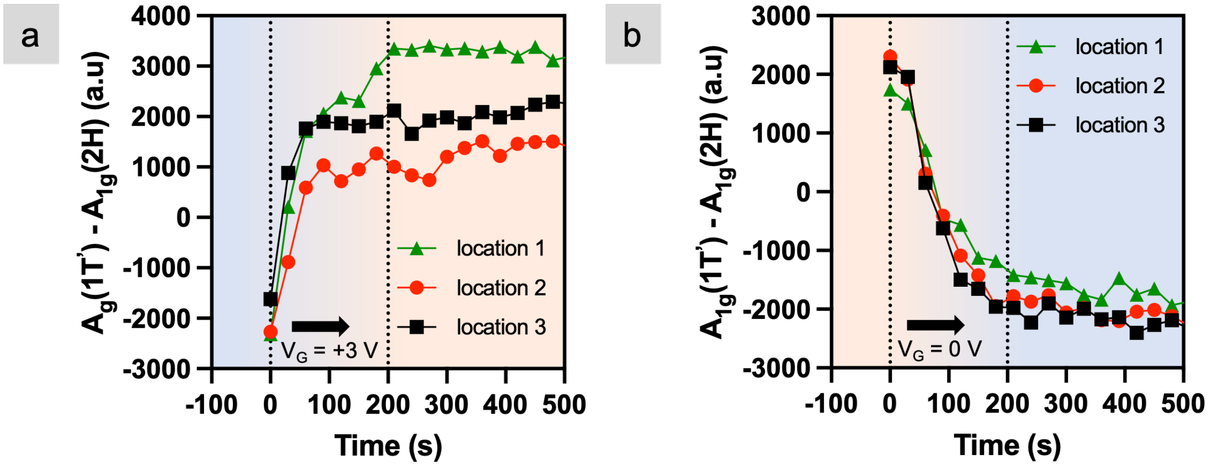

Figure S13: Dynamics of phase transition: time dependent Raman spectroscopy of (a) intrinsic 2H phase $MoTe_2$ device by applying V_{SG} = +3 V and (b) transformed 1T′ phase by applying V_{SG} = 0 V. Note that the three different datasets indicate three different locations on the same flake

14. EDL formation dynamics

As reported in section 9, the intrinsic phase transition time of the $MoTe_2$ is $\sim$ 200 s. The phase transition time will be a combination of the time required to either form or dissipate the double layer and the time for the atoms in the $MoTe_2$ to rearrange. Here, we show that the EDL formation on a supported $MoTe_2$ FET gated using a single-ion conductor is $\sim$ 20 s (shown in Supplementary Figure S14), which is an order magnitude smaller than the observed phase transition time of $\sim$ 200 s. Thus, the transition time is not limited by ion dynamics.

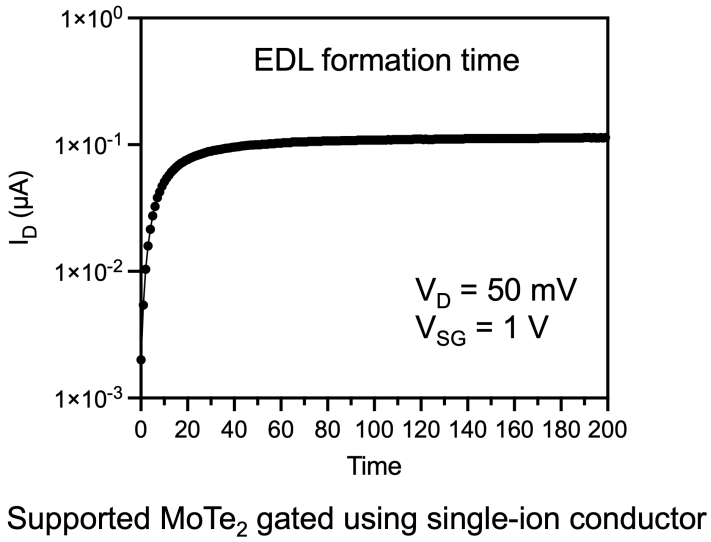

Figure S14: Time-dependent current of a supported $MoTe_2$ FET gated using a single-ion conductor at V_{SG} = +2 V and V_D = 50 mV.

15. Electrical characterization of the bare suspended $MoTe_2$ FET

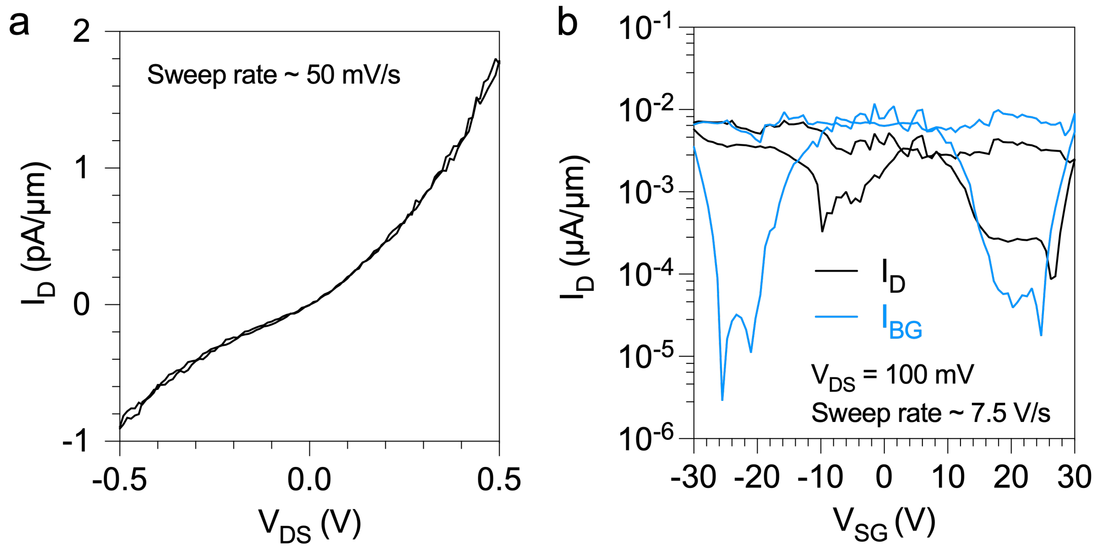

Figure S15: Electrical characterization of the suspended $MoTe_2$ FET before single ion conductor deposition. (a) Output measurement (b) back-gated transfer measurement. Note that the drain currents in the output measurements are in pA because we are likely inside the bandgap. The poor gate control in the transfer measurements is because of there is ~ 40 nm air gap and 90 nm of SiO_2 between the back gate and the channel. In addition, there is 60 nm of h-BN and 90 nm SiO_2 between the S/D and back gate contacts.

References

(1) Tan, Y.; Luo, F.; Zhu, M.; Xu, X.; Ye, Y.; Li, B.; Wang, G.; Luo, W.; Zheng, X.; Wu, N.; Yu, Y.; Qin, S.; Zhang, X. A. Controllable 2H-to-1T{'} phase transition in few-layer MoTe2. *Nanoscale* **2018**, *10*, 19964–19971.
